# Pepper leaf curl Lahore virus requires the DNA B component of Tomato leaf curl New Delhi virus to cause leaf curl symptoms

**DOI:** 10.1186/1743-422X-7-367

**Published:** 2010-12-13

**Authors:** Muhammad Shafiq, Shaheen Asad, Yusuf Zafar, Rob W Briddon, Shahid Mansoor

**Affiliations:** 1 Agricultural Biotechnology Division, National Institute for Biotechnology and Genetic Engineering (NIBGE), P O Box 577, Jhang Road, Faisalabad, Pakistan

## Abstract

**Background:**

Begomoviruses are whitefly-transmitted geminiviruses with genomes that consist of either two components (known as DNA A and DNA B) or a single component (homologous to the DNA A component of bipartite begomoviruses). Monopartite begomoviruses are often associated with a symptom-modulating DNA satellite (collectively known as betasatellites). Both bipartite and monopartite begomoviruses with associated satellites have previously been identified in chillies showing leaf curl symptoms in Pakistan.

**Results:**

**A **chilli plant (*Capsicum annum*) with chilli leaf curl disease symptoms was found to contain a begomovirus, a betasatellite and the DNA B component of *Tomato leaf curl New Delhi virus *(ToLCNDV). The begomovirus consisted of 2747 nucleotides and had the highest sequence identity (99%) *with Pepper leaf curl Lahore virus *(PepLCLV-[PK: Lah:04], acc. no. AM404179). *Agrobacterium*-mediated inoculation of the clone to *Nicotiana benthamiana*, induced very mild symptoms and low levels of viral DNA, detected in systemically infected leaves by PCR. No symptoms were induced in *Nicotiana tabacum *or chillies either in the presence or absence of a betasatellite. However, inoculation of PepLCLV with the DNA B component of ToLCNDV induced leaf curl symptoms in *N. benthamiana*, *N. tabacum *and chillies and viral DNA accumulated to higher levels in comparison to plants infected with just PepLCLV.

**Conclusions:**

Based on our previous efforts aimed at understanding of diversity of begomoviruses associated with chillies, we propose that PepLCLV was recently mobilized into chillies upon its interaction with DNA B of ToLCNDV. Interestingly, the putative rep-binding iterons found on PepLCLV (GGGGAC) differ at two base positions from those of ToLCNDV (GGTGTC). This is the first experimental demonstration of the infectivity for a bipartite begomovirus causing chilli leaf curl disease in chillies from Pakistan and suggests that component capture is contributing to the emerging complexity of begomovirus diseases in the region.

## Background

Viruses of the family *Geminiviridae *have circular, single-stranded (ss) DNA genomes and are divided into four genera based upon genome arrangement, host range and insect vectors. The most numerous, and economically the most destructive, are the whitefly-transmitted geminiviruses that are included in the genus *Begomovirus *[1,2]. Begomoviruses are transmitted by the whitefly *Bemisia tabaci *and exclusively infect dicotyledonous plants. They have emerged everywhere in the world where environmental conditions support large whitefly populations, and have become a major constraint in the production of food and fiber crops such as cassava, tomato, cucurbits, pepper, beans and cotton [3-5].

Chilli leaf curl disease (ChLCD) is an important factor limiting chilli production on the Indian subcontinent and is caused by begomoviruses [6-8]. Symptoms of the disease are severe leaf curl with cup-shaped, upward curling leaves, yellowing, and stunted plant growth. Previously chilli leaf curl betasatellite (ChLCB) has been identified in a large collection of chilli samples with leaf curl symptoms from all over the Pakistan [9]. A single species of betasatellite (ChLCB) was found associated with isolates showing geographical segregation and are similar to that reported earlier [6]. Chilli peppers often show symptoms similar to tomato leaf curl disease, such as yellowing, leaf curling, a reduction in leaf size and stunting. Since chilli and tomato crops overlap in the field, it is likely that chilli peppers may become infected with tomato begomoviruses. The disease was experimentally transmitted from infected to healthy chilli and tomato seedlings by the whitefly *Bemisia tabaci *[10,11]. Inoculated chilli plants developed typical symptoms of the disease. However, the inoculated tomato plants developed severe leaf curl symptoms similar to those of leaf curl disease of tomato caused by *Tomato leaf curl New Delhi virus *(ToLCNDV) [11,12]. Analysis of a large collection of chilli samples from Pakistan showed that diverse begomoviruses may infect chillies [7]. Recently another distinct begomovirus, *Pepper leaf curl Lahore *(PepLCLV), has been identified in chilli in Pakistan although the infectivity of the virus to chillies was not established experimentally [13].

Here we have characterised a further isolate of PepLCLV from chilli and have investigated its interaction with betasatellites and the DNA B component of ToLCNDV. We show that the virus requires the DNA B of ToLCNDV to infect plants and induce disease symptoms but its interaction with ChLCB is poor.

## Materials and methods

### Collection of virus infected plant samples

A chilli plant showing typical symptoms of begomovirus infection was observed in a field near Faisalabad, Pakistan, in 2004. A leaf sample with leaf curl symptoms and a leaf from an asymptomatic plant were collected. Samples were brought to the laboratory and stored at -80°C before extraction of DNA. This isolate was previously shown to harbour ChLCB ([PK:Fai69:04]; acc. no. AM279673) [9].

### Isolation, cloning and sequencing

Total DNA was extracted from symptomatic and asymptomatic chilli samples using the CTAB method [14]. Universal primers were used in PCR to amplify full-length begomovirus, betasatellite and alphasatellite molecules [15-17]. Two primer pairs, BC1F/BC1R [10], were used for the detection of DNA B of ToLCNDV. PCR amplified products of the expected sizes resulting from DNA extracted from the symptomatic leaf sample were cloned into the pTZ57R/T vector (Fermentas, Arlington, Canada) and a single clone, containing a potentially full-length begomovirus clone (~2800 nt), designated PGL1, was selected for further analysis. PGL1 was sequenced in both orientations with no ambiguities remaining (Macrogen, Korea). DNA sequences were assembled and analyzed with the aid of the Lasergene package of sequence analysis software (DNA Star Inc., Madison, WI, USA), and multiple sequence alignments were performed using Clustal X [18]. Phylogenetic trees were constructed using Clustal X (neighbor-joining method), displayed, manipulated and printed using Treeview [19]. Specific pairwise comparisons of all available sequences in the databases used the Pairwise Sequence Comparison (PASC) tool http://www.ncbi.nlm.nih.gov/sutils/pasc.

### *Agrobacterium*-mediated inoculation of plants

Standard methods were used to produce partial direct and tandem repeat constructs for *Agrobacterium*-mediated inoculation in the binary vector pGreen0092 [20]. After sequence confirmation of PGL1, a 1498 nt fragment encompassing the intergenic region was excised from with *Xba*I and *Eco*RI and subcloned into the *Xba*I-*Eco*RI sites of pGreen0092 to produce the clone pGPeA. The full-length insert of PGL1 was excised with *Xba*I and inserted into pGPeA at its unique *Xba*I site to yield clone icPGL1. Insert integrity and orientation were confirmed by digestion with *Eco*RI. This partial direct repeat of PGL1 was mobilized into *Agrobacterium tumefaciens *strain GV3103 by electroporation.

The infectivity of PGL1 was performed alone or in combination with the DNA B component of ToLCNDV [21], chilli leaf curl betasatellite (ChLCB-[PK:MC:97]; acc. no AJ316032; [6]) and cotton leaf curl Multan betasatellite (CLCuMB-[PK:Fai1:96]; AJ298903; [22]) in *Nicotiana benthamiana*, *N. tabacum *cv Samsun, and *Capsicum annum *cv Loungi by *Agrobacterium*-mediated inoculation. A total of 10 plants were inoculated for each plant species. Plants were kept at 25°C with 70% relative humidity and 16 h/day light in an insect-free greenhouse. Plants were examined daily for the appearance of symptoms.

## Results

### Detection of begomovirus components in chilli samples showing leaf curl symptoms

We have previously shown that chillies with leaf curl symptoms are associated with both monopartite begomoviruses along with a betasatellite as well as ToLCNDV [12]. Initially, to confirm the presence of a begomovirus in the symptomatic sample collected, diagnostic primer pairs were used in PCR amplifications with total nucleic acids extracted from the plant. Universal primers that amplify begomovirus DNA A, BegomoF and BegomoR, were used in PCR [17]. A PCR product of the expected size (approximately 2.8 kb) was amplified from the symptomatic chilli plant, and no amplification products of the expected size were obtained from healthy or asymptomatic chilli plants, confirming the association of a begomovirus with the disease. The DNA B of ToLCNDV was detected using primer pair BC1F/BC1R [10]. These primers are specific for the movement protein (MP) gene of ToLCNDV and gave an amplification product of approximately 850 nt, confirming the presence of ToLCNDV DNA B in the symptomatic plant. The presence of a betasatellite in samples was detected using the universal primer pair Beta01/Beta02 [16]. These primers produced a fragment of approximately 1350 nt and confirmed the presence of a betasatellite in the sample. The betasatellite was characterized earlier and shown to be an isolate of ChLCB [9].

### Analysis of the sequence of PGL1

**Figure 1:**
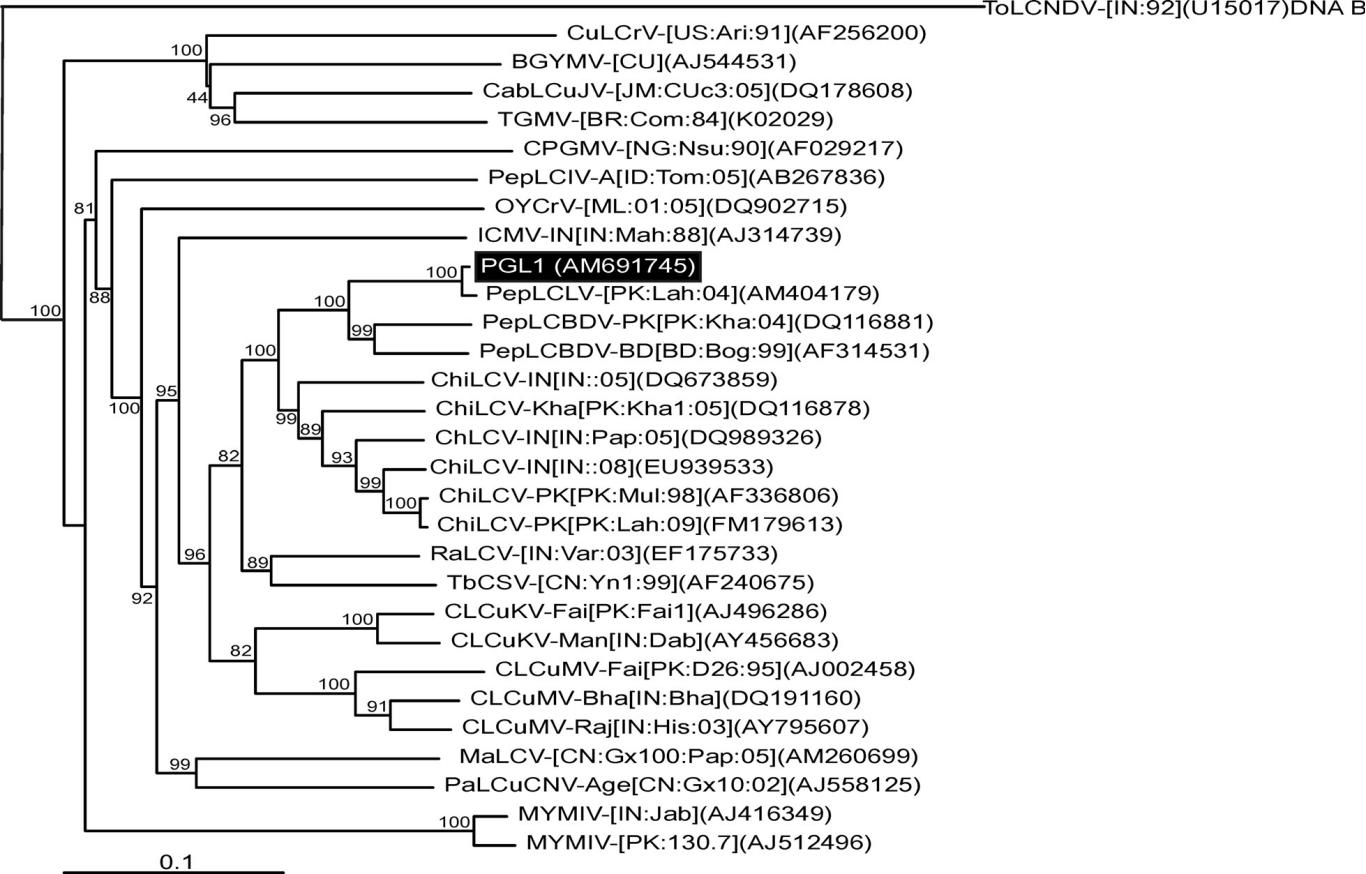
**Phylogenetic dendrograms based upon an alignment of selected complete sequences (or DNA A components) of begomoviruses**. The begomovirus (or DNA A component) sequences used for the alignment are *Bean golden yellow mosaic virus *(BGYMV), *Cabbage leaf curl Jamaica virus *(CabLCuJV), *Chili leaf curl virus *(ChLCV), *Cowpea golden mosaic virus *(CPGMV), *Cotton leaf curl Kokhran virus *(CLCuKV), *Cotton leaf curl Multan virus *(CLCuMV), *Cucurbit leaf crumple virus *(CuLCrV), *Indian cassava mosaic virus *(ICMV), *Malvastrum leaf curl virus *(MaLCV), *Mungbean yellow mosaic India virus *(MYMIV), *Okra yellow crinkle virus *(OYCrV), *Papaya leaf curl China virus *(PaLCuCNV), *Pepper leaf curl Bangladesh virus *(PepLCBDV), *Pepper yellow leaf curl Indonesia virus *(PepLCIV), *Pepper leaf curl Lahore virus *(PepLCLV), *Radish leaf curl virus *(RaLCV), *Tobacco curly shoot virus *(TbCSV) and *Tomato golden mosaic virus *(TGMV). The tree was arbitrarily rooted on the sequence of the DNA B component of *Tomato leaf curl New Delhi virus *(ToLCNDV). The database accession number in each case is given. Isolate and strain descriptors are as given in Fauquet *et al*. [23].

The sequence of the begomovirus clone PGL1 was determined to be 2747 nt in length and this sequence is available in the databases under accession number AM691745. Sequence comparisons revealed that the genome had the highest sequence identity (99%) with PepLCLV-[PK:Lah:04](AM404179) followed by 89% with PepLCuBDV-PK[PK:Kha:04](DQ116881). This indicates that PGL1 is an isolate of PepLCLV for which we propose the isolate descriptor PepLCLV-[PK:Fai:04] [23]. This conclusion is supported by a phylogenetic analysis which shows PGL1 to group with the only other isolate of PepLCLV for which a full-length sequence is available in the databases (PepLCLV-[PK:Lah:04]) and to be closely related to PepLCBDV (Figure 1).

**Figure 2:**
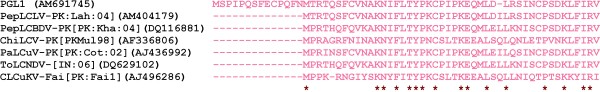
**Alignment of the N-terminal amino acid sequences of the Rep protein of PepLCLV (PGL1) clone with the sequences of other begomoviruses infecting chilli on the Indian subcontinent**. Gaps (-) were introduced into the sequences to optimize the alignment. Conserved sequences in the alignment are marked (*). The begomovirus (or DNA A component) sequences used for the alignment are *Chili leaf curl virus *(ChLCV), *Cotton leaf curl Kokhran virus *(CLCuKV), *Papaya leaf curl virus *(PaLCuV), *Pepper leaf curl Bangladesh virus *(PepLCBDV), *Pepper leaf curl Lahore virus *(PepLCLV), and *Tomato leaf curl New Delhi virus *(ToLCNDV). The database accession number in each case is given. Isolate and strain descriptors are as given in Fauquet *et al*. [23].

**Figure 3:**
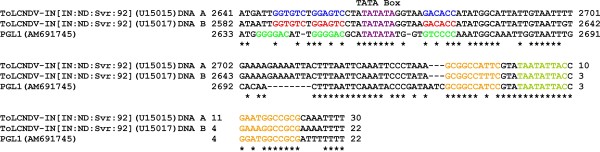
**Alignment of the intergenic region sequences of PepLCLV (PGL1), ToLCNDV DNA A and DNA B**. Gaps (-) were introduced into the sequences to optimize the alignment. Conserved sequences in the alignment are marked (*). The positions of the stem (highlighted in light orange) and conserved nonanucleotide sequences (highlighted in lime) of the predicted stem-loop structure, the TATA box of the Rep promoter (highlighted in violet) and predicted iterons (highlighted in dark green for PepLCLV, blue for ToLCNDV DNA A and red for ToLCNDV DNA B) are indicated.

The clone shows the typical genome organization of begomoviruses with two open reading frames (ORFs) in the virion-sense (V2 and V1) and four in the complementary-sense (C1, C2, C3 and C4) [2]. The main genetic features of this begomovirus sequence are given in Table 1. Among the six ORFs, a small difference was noted in the gene encoding the replication-associated protein (Rep). The sequence of the PepLCLV isolate determined here has the capacity to encode a Rep that is larger than that of the only other isolates of this virus characterised to date [13], as well as those of other begomoviruses. The Rep protein has a potential 14 amino acid leader sequence at the N-terminal end that is not present in closely related begomoviruses infecting pepper (Figure 2). The intergenic region (IR) consists of approximately 241 nucleotides and is similar to those of ToLCNDV isolates (Figure 3 Table 2). The IR contains a predicted stem-loop sequence with conserved nonanucleotide sequence (TAATATTAC) in the loop which can be found in the majority of geminiviruses characterized to date and marks the origin of virion-strand DNA replication [24]. Within the intergenic region, incomplete direct repeats of an iteron (GGGGAC) were detected adjacent to the TATA box of the Rep promoter. These sequences are species specific Rep binding motifs [25,26].

**Table 1:** Features of the begomovirus isolated from Capsicum annum

ORF*	Start codon (nucleotide coordinates)	Stop codon (nucleotide coordinates)	Predicted size of ORFs (nt)	Predicted size of protein (no. of amino acids)
V2	510	145	365	122
CP	1075	305	770	257
Rep	2651	1524	1127	376
TrAP	1621	1217	404	134
REn	1476	1072	404	134
C4	2452	2195	257	86

* Genes are indicated as coat protein (CP), replication-associated protein (Rep), transcriptional activator protein (TrAP), and replication enhancer (REn). The products encoded by ORFs V2 and C4 have yet to be named.

**Table 2:** Pairwise percent of nucleotide identities between the genomic components and amino acid sequence identities of encoded genes from the virus isolate PGL1 with the components and genes of selected other begomoviruses available in the databases.

Begomovirus	Complete sequence (percentage nucleotide sequence identity)	Intergenic region (percentage nucleotide sequence identity)	**Gene**^**#**^ (percentage amino acid sequence identity)					
			
			AV2	CP	REn	TrAP	Rep	AC4
ChLCV [11]*	84.0-87.2	77.3-88.3	87.9-92.4	94.4-98.4	75.8-91.7	82.6-97.7	75.2-91.0	39.3-92.9
CLCuMV [10]*	74.0-74.6	59.4-63.8	63.0-74.1	82.3-94.4	63.6-76.9	57.6-68.2	69.8-72.4	40.5-46.4
EACMV [10]*	69.6-69.9	62.4-64.0	62.1-62.9	75.4-76.2	68.9-70.5	63.6-65.2	53.6-65.8	26.0-27.3
PapLCV [5]*	72.3-87.0	61.9-85.9	69.1-90.5	92.5-97.6	73.5-90.9	70.5-95.5	61.3-91.0	40.5-94.0
PepLCV [4]*	74.0-86.7	51.7-80.4	75.0-91.4	78.1-98.0	66.7-90.9	71.2-91.7	69.6-75.8	34.5-35.7
PepLCBDV [3*]	88.6-89.4	78.7-85.8	89.7-92.2	94.8-96.0	90.2-95.4	93.9-97.7	90.7-93.5	92.9-92.9
PepLCLV [2]*	98.9-99.0	96.2-96.7	97.4-98.3	98.0-98.4	99.2-99.2	100	97.5-98.3	97.6-100
ToLCNDV [4]*	71.4-71.8	64.8-66.0	68.2-70.0	92.1-93.3	63.6-67.4	54.5-56.8	67.2-68.6	39.7-41.4
ToLCGV [7]*	77.8-78.5	73.5-75.1	85.0-86.7	78.6-78.6	79.5-81.8	87.9-90.9	74.4-75.6	34.5-36.9

* Numbers of sequences from the databases used in the comparisons.

^# ^Gene names are as indicated in Table 1.

### Infectivity and symptoms of PepLCLV

**Figure 4:**
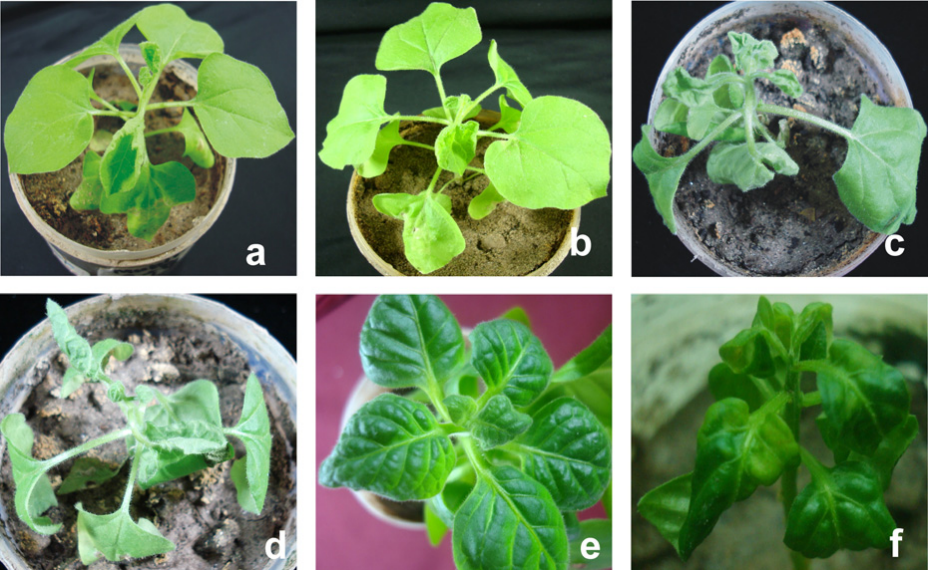
**Symptoms induced by PepLCLV clone PGL1 in *N. benthamiana, N. tabacum *and *C. annum***. **a) **An *N. benthamiana *plant infected with PepLCLV at 20 days post-inoculation (dpi) **b) **An *N. benthamiana *plant infected with PepLCLV and ChLCB at 20 dpi. **c) **A *N. benthamiana *plant infected with PepLCLV and ToLCNDV DNA B at 14 dpi. **d) **A *N. benthamiana *plant infected with PepLCLV, ChLCB and ToLCNDV DNA B at 14 dpi. **e) **A *N. tabacum *plant infected with PepLCLV and ToLCNDV DNA B at 30 dpi. **f) **A *C. annum *plant infected with PepLCLV and ToLCNDV DNA B at 40 dpi.

**Figure 5:**
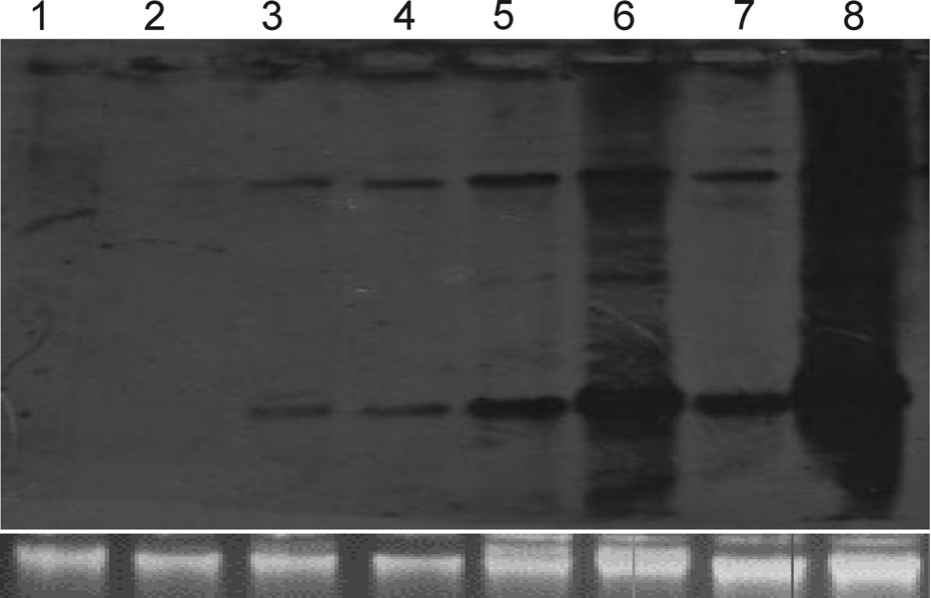
**Virus replication in systemic leaves of inoculated *N. benthamiana *plants probed with PGL1**. Plants were agroinoculated with PepLCLV (lane 1) PepLCLV and ChLCB (lane 2), PepLCLV, ChLCB and ToLCNDV DNA B (lanes 3,4), PepLCLV and ToLCNDV DNA B (lanes 5-7). The DNA sample in lane 9 was extracted from a chilli plant infected with PepLCLV collected in the field. Approximately 10 μg of total DNA was loaded per sample. A photograph of the genomic DNA on the ethidium bromide stained agarose gel is shown below the blot to confirm equal sample loading.

The infectivity of PepLCLV clone PGL1 was investigated in *N*. *benthamiana*, *N*. *tabacum *Samsun, and *C*. *annum *by *Agrobacterium*-mediated inoculation (Table 3). Inoculation of *N. benthamiana *with PepLCLV resulted in low infectivity (2/10) and infected plants exhibited very mild leaf curl symptoms (Figure 4). The virus was detected in systemic leaves by PCR with specific primers but was not detected by Southern blot hybridisation (Figure 5), indicating that only very low levels of virus DNA accumulated in infected plants. ChLCB was infectious to *N. benthamiana *with the helper virus *Cotton leaf curl Multan virus *(CLCuMV) and induced very severe leaf curl symptoms (Table 3), indicating that the ChLCB clone is infectious and capable of enhancing helper virus symptoms. However, when inoculated in the presence of ChLCB, PepLCLV also only induced very mild symptoms in *N. benthamiana *(Figure 4) and the virus levels in plants were below the detection threshold of Southern blot hybridisation (Figure 5). Inoculation of PepLCLV to *N. tabacum*, either alone or with ChLCB did not result in infection. In order to further study the interaction of PepLCLV with betasatellites, the clone was inoculated with another distinct betasatellite, CLCuMB [6]. However, inoculation with these betasatellites did not result in infection. Both CLCuMB and ChLCB were infectious to *N. benthamiana *with the helper virus CLCuMV (Table 3).

**Table 3:** Infectivity and symptoms induced by Pepper leaf curl Lahore virus

Plant species	Inoculum	Infectivity (plants infected/inoculated)					Symptoms
			
		Experiment					
			
		I	II	III	IV	Total	
*N. benthamiana*	PepLCLV	2/10	1/6	0/7	1/5	4/28	very mild leaf curling
	PepLCLV + ChLCB	1/10	0/6	1/7	0/5	2/28	very mild leaf curling
	PepLCLV + ToLCNDV DNA B	9/10	6/6	6/7	4/5	25/28	severe downward leaf curling
	PepLCLV + ToLCNDV DNAB + ChLCB	8/10	5/6	7/7	4/5	24/28	severe downward leaf curling
	PepLCLV + CLCuMB	0/10	-	-	-	0/10	no symptoms
	CLCuMV + ChLCB	4/5	5/6	-	-	9/11	severe leaf curling
	CLCuMV + CLCuMB	5/5	-	-	-	5/5	severe leaf curling

*N. tabacum*	PepLCLV + ChLCB	0/10	0/4	-	-	0/14	no symptoms
	PepLCLV + ToLCNDV DNA B	8/10	3/6	-	-	11/16	leaf curling

*C. annum*	PepLCLV + ChLCB	0/10	0/5	-	-	0/15	no symptoms
	PepLCLV + ToLCNDV DNA B	5/10	3/6	-	-	8/16	leaf curling

### PepLCLV *trans*-replicates ToLCNDV DNA B and induces leaf curl symptoms

Agroinoculation with partial repeats of PepLCLV along with the DNA B of ToLCNDV [21] induced leaf curl symptoms in *N*. *tabacum *Samsun, *N*. *benthamiana*, and *C. annum*. The symptoms in *N. benthamiana *consisted of severe upward leaf curling and severe stunting. Southern hybridization using PepLCLV as probe detected typical begomovirus replication intermediates (Figure 5). Virus levels accumulating in plants inoculated with PepLCLV alone were not detectable by Southern hybridization (Figure 5 lane 2). Inoculation of PepLCLV with ToLCNDV DNA B and ChLCB resulted in disease symptoms but the virus levels were lower (Figure 5 lanes 3 and 4) than in plants inoculated with PepLCLV and ToLCNDV DNA B (Figure 5 lanes 5-7). Symptoms in chillies and *N. tabacum *consisted of downward leaf curling and yellowing.

## Discussion

The geminiviruses are a rapidly emerging group of plant viruses, which can be attributed to various factors, including increased insect vector populations and the presence of alternative hosts. Though it was speculated that geminiviruses had the capacity to evolve rapidly in response to changes in their environment (such as alterations in cropping systems and/or population dynamics of the insect vector), there are few studies documenting geminivirus evolution. The success in the Old World of begomoviruses that associate with betasatellites appears to be due to the ability of betasatellites to be replicated by several distinct begomoviruses [27]. Thus, the ability of begomoviruses to interact with diverse betasatellites, the mobilization of begomovirus components from alternate hosts and recombination among begomoviruses or associated satellites has been documented as the major driving force in rapid emergence and resistance breakdown by begomovirus-betasatellite complexes [5]. There are numerous reports documenting mixed infection of geminiviruses. For example, in cassava, infection by two distinct begomovirus species resulted in a severe disease due to synergism [28]. Interestingly, ToLCNDV, a bipartite begomovirus has been consistently detected in several hosts in the Indian subcontinent and suggest that the virus has flexibility in its interaction with other begomovirus components that may help virus to expand host range. We have previously shown that ToLCNDV interacts with ChLCB under field conditions that result in severe symptoms [12]. Another study showed that *Tomato leaf curl Gujarat virus *captured the DNA B component of ToLCNDV, resulting in a virus capable of inducing more severe disease symptoms [29,30]. Our recent analysis has shown that ToLCGV may exist without a DNA B in some weeds (M. Mubin, manuscript in preparation) which suggests that ToLCGV was mobilized from a weed into tomato upon its interaction with the DNA B of ToLCNDV. Thus, it appears that component capture during mixed infection, probably in weed hosts, may result in viruses with enhanced virulence to crop plants.

Geminivirus genomes replicate by a rolling circle mechanism which is initiated by the virus-encoded replication-associated protein (Rep) [31]. Rep is a sequence specific DNA binding protein which recognises and binds to repeated sequences, known as iterons, in the intergenic region immediately upstream of a hairpin structure that contains the ubiquitous (for geminiviruses) nonanucleotide sequence (TAATATTAC). Rep then initiates replication by nicking in the nonanucleotide sequence. The DNA A and DNA B components of bipartite begomoviruses have the same iteron sequences, thereby ensuring that the DNA A-encoded Rep may initiate replication of both components; maintaining the integrity of the split genome. However, mutational analyses and sequencing of field isolates suggests that begomoviruses may tolerate some sequence variation in iteron sequences without deleterious effects on Rep recognition. Here we show that the predicted iteron sequence of PepLCLV (GGGGAC) differs at two base positions from the iteron sequence of ToLCNDV (GGTGTC; Figure 5). Thus, it appears that the first two or three bases may be more important in iteron recognition by Rep. An interesting finding of the study is that PepLCLV isolate examined here has no, or at least only limited, ability to trans-replicate betasatellites. This contrasts with the results of Tahir *et al*. [13], who showed the association of ChLCB with another isolate of PepLCLV from Pakistan. Our analysis suggests that the putative Rep protein of the virus has an N-terminal leader sequence (Figure 2) which may be important in the inability of the virus to trans-replicate betasatellites. However, in the absence of any evidence this remains a hypothesis that requires confirmation by mutagenesis. The other possibility may be that there are natural variants of PepLCLV that lack leader peptide in the Rep protein that may be able interact with betasatellites. Since our efforts to characterize begomoviruses in recent samples is not exhaustive, it will not be surprising if another begomovirus capable of interaction with betasatellite is present in naturally infected chillies.

Chilli leaf curl disease (ChLCD) is an important factor limiting chilli production across Pakistan and India. The genomes of begomoviruses are either bipartite (with two genomic components known as DNA A and DNA B) monopartite (with a genome consisting of only a homolog of the DNA A component) or monopartite associated with a symptom determining satellite (collectively known as betasatellites). All three types have been previously identified in chillies [9,10,12]. The full-length genome of a begomovirus associated with ChLCD originating from the Punjab (Pakistan), PGL1 was cloned and shown to consist of 2747 nucleotides. Sequence comparisons showed that the genome had the highest sequence identity (99%) with Pepper leaf curl Lahore virus (PepLCLV-[PK:Lah:04]) indicating that it represents an isolate of PepLCLV based on the 89% species demarcation threshold for begomoviruses [23]. *Agrobacterium*-mediated inoculation of the clone to *N. benthamiana *induced only very mild symptoms. Inoculation of the clone with a betasatellite previously isolated from a ChLCD affected plant gave similarly mild symptoms and virus levels were not detectable by Southern hybridization. However, inoculation with the DNA B component of ToLCNDV induced symptoms typical of ChLCD in *N. benthamiana*, *N. tabacum *and *C. annum*. These results suggest that the virus characterised here may be bipartite. A surprising finding is that the levels of viral DNA were lower in plants inoculated with PepLCBDV, ToLCNDV DNA B and ChLCB in comparison to plants inoculated in the absence of ChLCB (Figure 5). This is the first experimental demonstration of infectivity for a bipartite begomovirus causing ChLCD.

These results presented here demonstrate that ChLCD in Pakistan may be caused by a bipartite variant of PepLCLV, which is associated with a DNA B component related to ToLCNDV DNA B, in addition to a monopartite variant of PepLCLV which associates with a betasatellite (ChLCB) [13]. The difference in the ability of the two PepLCLV isolates to interact with ChLCB may be due to the presence, in the isolate characterised here, of additional N-terminal amino acid sequences of Rep. However, since we as yet do not fully understand the mechanism of interaction of begomovirus-encoded Rep with betasatellites to initiate satellite replication (betasatellites lack the iteron sequences encoded by their helper viruses [27]), this will require experimental confirmation. Despite the differences in the predicted iteron sequences of PepLCLV and ToLCNDV, PepLCLV has the ability to trans-replicate ToLCNDV DNA B and induce ChLCD in experimental hosts and chilli. This is the first experimental demonstration of Koch's postulates using cloned viral DNA components for a bipartite begomovirus causing ChLCD. The bipartite PepLCV has some residual ability to interact with betasatellites although the presence in plants of both a DNA B and a betasatellites appears to reduce virus titre and symptom severity, suggestive of interference. The nature of this interference will be the focus of our future studies since this may provide a novel mechanism of obtaining resistance to the viruses causing ChLCD. The complex nature of ChLCD across the Indian sub-continent, which has been shown to be caused by several bipartite and monopartite, betasatellite-associated begomoviruses will be a challenge for the development of resistant varieties either by conventional or non-conventional means. The high yield losses resulting from ChLCD are threatening chilli cultivation and are forcing farmers in some areas to grow other crops.

## Competing interests

The authors declare that they have no competing interests.

## Authors' contributions

MS performed the experiments. MS, SA, YS, RWB and SM were involved in data analysis. SA, YS, RWB and SM provided overall direction and experimental design. RWB and SM wrote the manuscript. All authors read and approved the final manuscript.
